# User-Centred Healing-Oriented Conditions in the Design of Hospital Environments

**DOI:** 10.3390/ijerph15102140

**Published:** 2018-09-28

**Authors:** Mateja Dovjak, Masanori Shukuya, Aleš Krainer

**Affiliations:** 1 Chair of Buildings and Constructional Complexes, Faculty of Civil and Geodetic Engineering, University of Ljubljana, 1000 Ljubljana, Slovenia; 2 Faculty of Health Sciences, University of Ljubljana, Zdravstvena pot 5, 1000 Ljubljana, Slovenia; 3 Department of Restoration Ecology and Built Environment, Tokyo City University, Yokohama 224-8551, Japan; shukuya@tcu.ac.jp; 4 Institute of Public and Environmental Health, 1000 Ljubljana, Slovenia; ales.krainer@stavba2020.si

**Keywords:** healing conditions, hospital environment, burn patient, thermodynamic response, user-centred cyber-physical system

## Abstract

Design approaches towards energy efficient hospitals often result in a deteriorated indoor environmental quality, adverse health and comfort outcomes, and is a public health concern. This research presents an advanced approach to the design of a hospital environment based on a stimulative paradigm of healing to achieve not only healthy but also comforting conditions. A hospital room for severely burn patient was considered as one of the most demanding spaces. The healing environment was designed as a multi-levelled, dynamic process including the characteristics of users, building and systems. The developed integral user-centred cyber-physical system (UCCPS) was tested in a test room and compared to the conventional system. The thermodynamic responses of burn patients, health care worker and visitor were simulated by using modified human body exergy models. In a healing environment, UCCPS enables optimal thermal balance, individually regulated according to the user specifics. For burn patient it creates optimal healing-oriented conditions with the lowest possible human body exergy consumption (hbExC), lower metabolic thermal exergy, lower sweat exhalation, evaporation, lower radiation and convection. For healthcare workers and visitors, thermally comfortable conditions are attained with minimal hbExC and neutral thermal load on their bodies. The information on this is an aid in integral hospital design, especially for future extensive renovations and environmental health actions.

## 1. Introduction

Present day extensive building renovations and constructions are going in the wrong direction towards unilateral measures oriented towards highly energy efficient buildings. Thermally optimized insulation and air-tightened building envelopes together with highly energy efficient heating, ventilation and air-conditioning (HVAC) systems often result in deteriorated indoor environmental quality (IEQ).

The problem of deteriorated IEQ in buildings was detected in the 1970s and coincided with the beginning of the energy crisis and interventions against it. The association between IEQ and health was confirmed in 1983, when it was estimated that up to 30% of new and renovated buildings worldwide may be related to an unhealthy indoor environment [1]. One of the most common health outcomes, evidenced by research studies, is sick building syndrome (SBS). It is described as non-specific symptoms associated with acute discomfort that appears during time spent in a building, but no specific illness or cause can be identified. Unlike SBS, building-related illness (BRI) is defined as symptoms of diagnosable illnesses that are identified and can be attributed directly to airborne building contaminants [2]. A typical example of BRI is aspergillosis that presents the possible health effect of exposure to damp buildings with *Aspergillus* mould [3].

In today’s era of one-sided renovations directed towards minimized building energy use, the situation of deteriorated IEQ has become even worse. The impacts of energy renovation on health and comfort outcomes have been highlighted by numerous researchers and pointed out by building users and inspectors [4,5]. In order to reduce building ventilation losses, the ventilation rate is often designed on the physiological minimal values, which are also permitted by national legislation (e.g., [6]). This, however, results in inadequate indoor air quality with higher indoor carbon dioxide concentrations. Higher indoor carbon dioxide concentrations have negative impact on human decision making performance and cause increased health symptoms and poorer perceived air quality [7]. Similar findings were made in a study on Slovak residential buildings, where it was clearly demonstrated that “energy renovation was associated with lower occupant satisfaction with indoor air quality” [4] (p. 363). Energy-efficiency retrofits as an overlooked public health concern were also criticized by Manuel [5] in an article on avoiding health pitfalls of home energy-efficiency retrofits: “Inspections have uncovered several instances of hazardous conditions created or worsened by retrofits, which serve as reminders of the need for care to ensure that home renovations don’t cause more problems than they cure” [5] (pp. A76–A79).

Our paper focuses on hospitals, with two leading motives. First, hospitals are considered as priority environments in building renovation strategies and plans for the reason of being the “largest non-residential buildings that consume 10% of final energy use in non-residential buildings in Europe” [8] (p. 37). Second, a current one-sided approach of the design of hospital buildings and HVAC systems does not follow the basic rules of engineering design, starting from the needs and demands of individual users. A hospital environment presents a highly demanding indoor environment, where needs and demands for the definition of environmental parameters are based on user specifics, health status, hygienic demands, specific activities and procedures. For example, the recommended range of operative temperature for newborns is from 35 °C (for 1 kg birth weight) to 32 °C (more than 2.5 kg of birth weight); the required air temperature for a ward for severe burn injuries is up to 32 °C and relative humidity is up to 95%; the required air temperature for cardiac surgery is 17 °C, corneal surgery 18–24 °C and paediatric surgery 30 °C [9,10]. A design approach to a hospital environment that does not take into account the needs and demands of specific user, especially vulnerable one, might result in inadequate indoor conditions, not only for patients but also for health care workers and visitors.

According to epidemiological studies, the negative consequences of insufficient IEQ in a hospital environment include deteriorated patient treatment outcome, increased hospitalization, lower level of productivity and increased absenteeism of staff [11]. Moreover, much higher prevalence of SBS was demonstrated in hospital environment than in other public buildings. The indicated prevalence of SBS was from 63–86% in hospital environment and from 20–50% in other public buildings [12,13,14]. SBS is a health outcome caused by exposure to numerous health risk factors and their interactions. However, the environmental parameters related to user comfort and building ventilation were identified as being the most important health risk factors for SBS. Namely, thermal comfort conditions in existing hospital environment are regulated by conventional HVAC systems (i.e., radiator, convector, air-conditioning (AC) device), which do not allow individual and dynamical temperature control. Additionally, ventilation is usually performed by central systems with air as a carrier.

Transition from an existing design practice to novel approach strategies is needed, where the creation of optimal and healing conditions for individual users are the priority. The origin of the healing environment principle is in the Anglo-Saxon word “haelen”, and means “to be or become whole” [15]. However, the original concept of a healing environment was developed by Florence Nightingale and means “to manipulate the environment to be therapeutic” [16]. Nightingale outlined the requirements of the healing environment to optimize the capacity of a patient to recover, including “quiet”,” warmth”, “clean air”, “light” and “good diet” [16]. All outlined requirements coincide today with well-known indicators for IEQ: thermal comfort, acoustic comfort, visual comfort, and non-image forming effects, indoor air quality, ergonomics, and universal design.

In the 1960s, healing environments were linked to evidence-based design, developed on a concept with a strong scientific base [15,17]. Since then, numerous studies have been undertaken, revealing the importance of healing environments for patients, staff and visitors. Benefits of healing environment are associated with improved medical treatment and healthcare process of patient as well as increased productivity and perceived comfort of other users. The role of creating of healing environment in the critical care units was emphasized by Stichler [18], who claimed: “physical healthcare environment can make a difference in how quickly the patient recovers from or adapts to specific acute and chronic conditions”. Devlin and Arneill [19] and Ulrich et al. [20] included in the definition also the relationship between patient and staff: a “healing environment is as a place where the interaction between patient and staff produces positive health outcomes within the physical environment”. Therefore, it is necessary to include evidence-based design as an integral part of every design, especially in specific environments such as hospitals.

Based on the systematic literature review by Dijkstra [21], physical environmental stimuli, as key elements of a healing environment, were defined and can be classified as ambient, architectural or interior design features. Dijkstra [21] highlighted the importance of reducing the effects of negative environmental stimuli or adding positive stimuli to environments for promoting health and well-being. In addition, the impact of physical environmental factors of heating environments on users was reviewed by Huisman et al. [22]. Besides the elements of the physical environment, Malkin [15] stressed that a psychologically supportive organizational culture also presents an important aspect of a healing environment. Despite numerous review studies, there is no integral design morphology of a healing environment.

The purpose of our research is to present the significance of an advanced approach to the design of hospital environment, based on the stimulative paradigm of healing and not only for healthy and comforting conditions. As an example, a hospital room for burn patient is considered one of the most demanding spaces. Regulation demands for a burn patient room [9] that “room air temperature has to be up to 32 °C and relative humidity up to 95%” [9,23,24]. “Mortality, morbidity and hospitalization can be significantly decreased with optimal environmental conditions” [23,24].

The first objective of our research was to present the design morphology of a healing environment, which is based on the needs and demands of individual users. The second objective was to develop and test the innovative user-centred cyber-physical system (UCCPS) that enables the achievement of optimal and healing conditions for patients and comfortable conditions for other users. The advanced approach presented can be an aid when preparing recommendations for environmental health policies and strategies.

## 2. Materials and Methods

### 2.1. Design Morphology of a Healing Environment

Linked up morphology for the design of living and working environment was devised based on a healing environment principle. Our work continues the design philosophy started by Intelligent Energy Europe project proposal, key action (CIP-IEE-PROMO-P) [25], where environmental conditions go beyond neutral comfort conditions. For the development of the morphology of a healing environment and system, a room for burn patients, as a case of an extremely demanding environment, was chosen. A healing environment was designed as multi-levelled and dynamic process and included users, with defined characteristics of the building, HVAC systems and control, i.e., integral user-centred cyber-physical system. Influential design parameters and their reciprocal influences were selected by using evidence-based design. All influential parameters were included in the matrix, presenting a tool for the simultaneous consideration of parameters, identification of their reciprocal influences, and definition of measures. At the level of building, we analysed active zones, building envelope, building systems, building geometry and orientation at the specific location. Individual characteristics were defined at the user level, which presents a guide for the definition of needs and demands for healing conditions and also the requirements for the design of active spaces. In the sequel, an integral UCCPS was designed. It enables to achieve healing and optimal conditions for a patient, and comfortable conditions for other users. Its functioning was tested in the test and model room and compared to the conventional system. Our research questions for the testing phase were: 1. Why is the creation of the required environmental conditions for burn patient important? 2. Has the selected type of the heating and cooling system impacted on thermodynamic response of burn patient? 3. Is it possible to actively regulate conditions in healing environment by considering the needs, demands and characteristics of individual users?

### 2.2. Experimental Part

**Figure 1:**
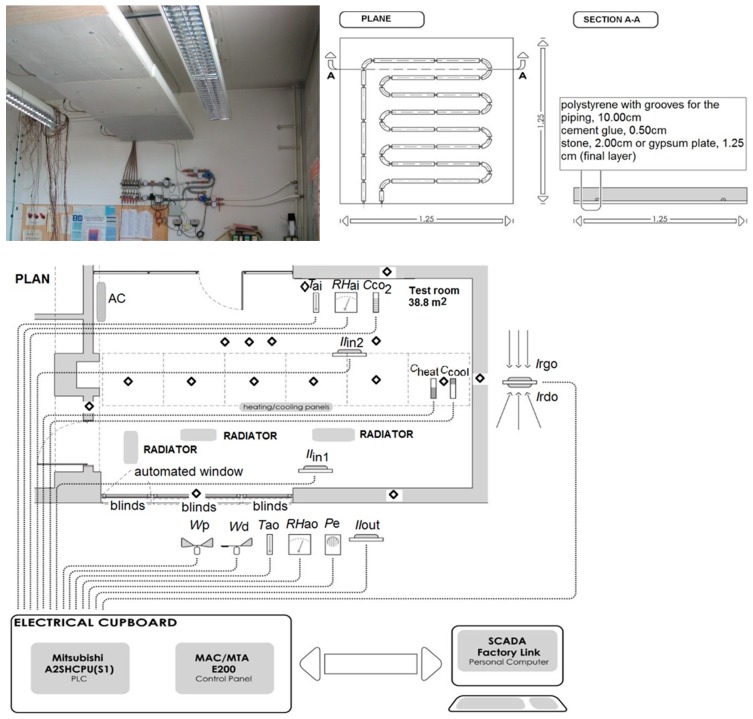
Test room with conventional and user-centred cyber-physical system (UCCPS) system, sensor position. Abbreviations: *T*_ai_, temperature of indoor air; *T*_ao_, temperature of outdoor air; *RH*_ai_, relative humidity of indoor air; *RH*_ao_, relative humidity of outdoor air; *Il*_in1_ *& Il*_in2_, internal work plane illumination; *Il*_out_, external illumination; *C*_${\mathrm{CO}}_{2}$_, concentration of CO_2_; *I*_rgo_, direct solar radiation; *I*_rdo_, reflected solar radiation; *W*_p_, wind speed; *W*_d_, wind direction; *P*_e_, precipitation detection; *C*_heat_, energy use for heating; *C*_cool_, energy use for cooling.

A combination of experimental monitoring of environmental conditions and simulation of thermodynamic responses of the human body was carried out in the test room and model room for burn patients (7.5 × 5.0 × 4.0 m). The test room is located at the Faculty of Civil and Geodetic Engineering, University of Ljubljana (latitude 46.05°, longitude 14.51°). The room is equipped with a UCCPS and conventional system (Figure 1). The UCCPS consist of 9 m^2^ low-temperature-heating and high-temperature-cooling ceiling radiative panels connected to the control and information system. The conventional system consists of three oil–filled electric heaters type Heller (230 V/50 Hz, 2000 W) and a split system with indoor unit for cooling. Humidification was performed by room humidifiers. A combination of monitoring of environmental conditions and simulation of thermodynamic responses of three individual users of active space was performed in connection with the used heating and cooling system and the characteristics of the building envelope.

The required conditions for the burn patient room were created with both systems. The selected monitoring parameters were indoor air temperature and relative air humidity (*T*_ai_, *RH*_ai_), outdoor air temperature and relative air humidity (*T*_ao_, *RH*_ao_), surface temperatures, black globe temperature, and temperature of the medium in panels. For the analysis of the user’s thermodynamic response to different environmental conditions, the assumptions were: *T*_ai_, mean radiant temperature (*T*_mr_) and *RH*_ai_ varied in the range 15–35 °C and 30–96%, respectively.

Three users (burn patient, health care worker and visitor) were selected for the simulation of thermodynamic response of the human body. From the comprehensive literature review (presented in the section Definition of specific user needs and demands), characteristics for a severely burned patient were chosen: 80% TBSA (percentage of the total body surface area that is affected by a burn), hypermetabolic state (2 met), hypothermia (body core temperature *T*_cr_ 35.5 °C, skin temperature *T*_sk_ 37.0 °C). Characteristics for health care workers were: metabolic rate 1.1 met, clothing insulation 0.6 clo. Characteristics for visitors were: 2.0 met, 0.6 clo. *T*_sk_ and *T*_cr_ were constant for the calculations for burn patient, and were changeable for visitors and health care workers. *T*_cl_ was calculated on the basis of experimental conditions.

Individual thermodynamic responses to environmental conditions were analysed by a human body exergy model developed by Shukuya et al. [26]. The model was built on the characteristics of an average healthy subject exposed to normal environmental conditions. For the purpose of our study, the model was modified according to the characteristics of three individual virtual users (burn patient, healthcare worker and visitor), exposed to various experimental conditions. The model room had the same characteristics as the test room. In the model room with a conventional system, *T*_ai_ = *T*_mr_ = *T*_o_ was assumed and in the room with UCCPS *T*_ai_ ≠ *T*_mr_ ≠ *T*_o_ was assumed.

### 2.3. Setting Thermal Exergy Balance of Burn Patient Body

The thermal exergy balance of human body [27] was derived by combining the water balance equation, energy balance equation and entropy balance equation under steady-state conditions. All of them are the resultant equations of the mathematical operations described in [27], together with the environmental temperature for exergy calculation, which in our case is outdoor air temperature. The thermodynamic system of the human body consists of a core and a shell (i.e., two-node model) and is positioned in the environment of the model room for burn patient. The general form of the exergy balance equation for the human body as a system is represented in Equation (1). If an overall investigation of the human body exergy balance is made together with space-heating or cooling system’s exergy balance, the environmental temperature must be the same for both the human body and the space heating or cooling system (indoor air temperature).

[Exergy input] − [Exergy consumption] = [Exergy stored] + [Exergy output](1)

The exergy inputs of the human body exergy balance (Equation (1)) consist of the following components:

(2) $$\begin{array}{l} \mathrm{Metabolic}\;\mathrm{thermal}\;\mathrm{exergy}\;\mathrm{rate}=[\mathrm{Warm}\;\mathrm{exergy}\;\mathrm{generated}\;\mathrm{by}\;\mathrm{metabolism}]+\\ \left[\mathrm{Warm}\;\mathrm{and}\;\mathrm{wet}\;\mathrm{exergies}\;\mathrm{of}\;\mathrm{the}\;\mathrm{liquid}\;\mathrm{water}\;\mathrm{generated}\;\mathrm{in}\;\mathrm{the}\;\mathrm{core}\;\mathrm{by}\;\mathrm{metabolism}\right]\\ +[\mathrm{Warm}/\mathrm{cool}\;\mathrm{and}\;\mathrm{wet}/\mathrm{dry}\;\mathrm{exergies}\;\mathrm{of}\;\mathrm{the}\;\mathrm{sum}\;\mathrm{of}\;\mathrm{liquid}\;\mathrm{water}\;\mathrm{generated}\;\mathrm{in}\;\mathrm{the}\\ \mathrm{shell}\;\mathrm{by}\;\mathrm{metabolism}\;\mathrm{and}\;\mathrm{dry}\;\mathrm{air}\;\mathrm{to}\;\mathrm{let}\;\mathrm{the}\;\mathrm{liquid}\;\mathrm{water}\;\mathrm{disperse}]+[\mathrm{Warm}\;\mathrm{exergy}\\ \mathrm{stored}\;\mathrm{in}\;\mathrm{the}\;\mathrm{core}\;\mathrm{and}\;\mathrm{the}\;\mathrm{shell}] \end{array}$$

(3) $$\begin{array}{l} \mathrm{Exergy}\;\mathrm{transferred}\;\mathrm{by}\;\mathrm{radiation},\;\mathrm{absorbed}=[\mathrm{Warm}/\mathrm{cool}\;\mathrm{radiant}\;\mathrm{exergy}\;\mathrm{absorbed}\\ \mathrm{by}\;\mathrm{the}\;\mathrm{whole}\;\mathrm{of}\;\mathrm{skin}\;\mathrm{and}\;\mathrm{clothing}\;\mathrm{surfaces}] \end{array}$$

(4) $$\begin{array}{l} \mathrm{Convective}\;\mathrm{exergy},\;\mathrm{absorbed}=[\mathrm{Warm}/\mathrm{cool}\;\mathrm{convective}\;\mathrm{exergy}\;\mathrm{by}\;\mathrm{absorbed}\;\mathrm{from}\\ \mathrm{the}\;\mathrm{whole}\;\mathrm{of}\;\mathrm{skin}\;\mathrm{and}\;\mathrm{clothing}\;\mathrm{surfaces}\;\mathrm{into}\;\mathrm{the}\;\mathrm{surrounding}\;\mathrm{air}] \end{array}$$

(5) $$\begin{array}{l} \mathrm{Sum}\;\mathrm{of}\;\mathrm{exergies}\;\mathrm{contained}\;\mathrm{by}\;\mathrm{the}\;\mathrm{inhaled}\;\mathrm{humid}\;\mathrm{air}=[\mathrm{Warm}/\mathrm{cool}\;\mathrm{and}\;\mathrm{wet}/\mathrm{dry}\\ \mathrm{exergies}\;\mathrm{of}\;\mathrm{the}\;\mathrm{inhaled}\;\mathrm{humid}\;\mathrm{air}] \end{array}$$

The exergy outputs of Equation (1) consist of the following components:

(6) $$\begin{array}{l} \mathrm{Exhalation}\;\mathrm{and}\;\mathrm{evaporation}\;\mathrm{of}\;\mathrm{sweat}=[\mathrm{Warm}\;\mathrm{and}\;\mathrm{wet}\;\mathrm{exergies}\;\mathrm{of}\;\mathrm{the}\;\mathrm{exhaled}\\ \mathrm{humid}\;\mathrm{air}]+[\mathrm{Warm}/\mathrm{cool}\;\mathrm{exergy}\;\mathrm{of}\;\mathrm{the}\;\mathrm{water}\;\mathrm{vapour}\;\mathrm{originating}\;\mathrm{from}\;\mathrm{the}\;\mathrm{sweat}\\ \mathrm{and}\;\mathrm{Wet}/\mathrm{dry}\;\mathrm{exergy}\;\mathrm{of}\;\mathrm{the}\;\mathrm{humid}\;\mathrm{air}\;\mathrm{containing}\;\mathrm{the}\;\mathrm{evaporated}\;\mathrm{water}\;\mathrm{from}\;\mathrm{the}\\ \mathrm{sweat}] \end{array}$$

(7) $$\begin{array}{l} \mathrm{Exergy}\;\mathrm{transferred}\;\mathrm{by}\;\mathrm{radiation},\;\mathrm{discharged}=[\mathrm{Warm}/\mathrm{cool}\;\mathrm{radiant}\;\mathrm{exergy}\\ \mathrm{discharged}\;\mathrm{from}\;\mathrm{the}\;\mathrm{whole}\;\mathrm{of}\;\mathrm{skin}\;\mathrm{and}\;\mathrm{clothing}\;\mathrm{surfaces}] \end{array}$$

(8) $$\begin{array}{l} \mathrm{Exergy}\;\mathrm{transferred}\;\mathrm{by}\;\mathrm{convection},\;\mathrm{discharged}=[\mathrm{Warm}/\mathrm{cool}\;\mathrm{exergy}\;\mathrm{transferred}\;\mathrm{by}\\ \mathrm{convection}\;\mathrm{from}\;\mathrm{the}\;\mathrm{whole}\;\mathrm{of}\;\mathrm{skin}\;\mathrm{and}\;\mathrm{clothing}\;\mathrm{surfaces}\;\mathrm{into}\;\mathrm{the}\;\mathrm{surrounding}\;\mathrm{air}] \end{array}$$

Considering the above components, the extensive human body exergy balance is presented as follows:

(9) $$\begin{array}{l} {}[\mathrm{Warm}\;\mathrm{exergy}\;\mathrm{generated}\;\mathrm{by}\;\mathrm{metabolism}]+[\mathrm{Warm}/\mathrm{cool}\;\mathrm{and}\;\mathrm{wet}/\mathrm{dry}\;\mathrm{exergies}\;\mathrm{of}\;\mathrm{the}\\ \mathrm{inhaled}\;\mathrm{humid}\;\mathrm{air}]+[\mathrm{Warm}\;\mathrm{and}\;\mathrm{wet}\;\mathrm{exergies}\;\mathrm{of}\;\mathrm{the}\;\mathrm{liquid}\;\mathrm{water}\;\mathrm{generated}\;\mathrm{in}\;\mathrm{the}\\ \mathrm{core}\;\mathrm{by}\;\mathrm{metabolism}]+[\mathrm{Warm}/\mathrm{cool}\;\mathrm{and}\;\mathrm{wet}/\mathrm{dry}\;\mathrm{exergies}\;\mathrm{of}\;\mathrm{the}\;\mathrm{sum}\;\mathrm{of}\;\mathrm{liquid}\;\mathrm{water}\\ \mathrm{generated}\;\mathrm{in}\;\mathrm{the}\;\mathrm{shell}\;\mathrm{by}\;\mathrm{metabolism}\;\mathrm{and}\;\mathrm{dry}\;\mathrm{air}\;\mathrm{to}\;\mathrm{let}\;\mathrm{the}\;\mathrm{liquid}\;\mathrm{water}\;\mathrm{disperse}]+\\ {}[\mathrm{Warm}/\mathrm{cool}\;\mathrm{radiant}\;\mathrm{exergy}\;\mathrm{absorbed}\;\mathrm{by}\;\mathrm{the}\;\mathrm{whole}\;\mathrm{of}\;\mathrm{skin}\;\mathrm{and}\;\mathrm{clothing}\;\mathrm{surfaces}]-\\ {}[\mathrm{Exergy}\;\mathrm{consumption}\;\mathrm{valid}\;\mathrm{only}\;\mathrm{for}\;\mathrm{thermoregulation}]=[\mathrm{Warm}\;\mathrm{exergy}\;\mathrm{stored}\;\mathrm{in}\\ \mathrm{the}\;\mathrm{core}\;\mathrm{and}\;\mathrm{the}\;\mathrm{shell}]+[\mathrm{Warm}\;\mathrm{and}\;\mathrm{wet}\;\mathrm{exergies}\;\mathrm{of}\;\mathrm{the}\;\mathrm{exhaled}\;\mathrm{humid}\;\mathrm{air}]+\\ {}[\mathrm{Warm}/\mathrm{cool}\;\mathrm{exergy}\;\mathrm{of}\;\mathrm{the}\;\mathrm{water}\;\mathrm{vapour}\;\mathrm{originating}\;\mathrm{from}\;\mathrm{the}\;\mathrm{sweat}\;\mathrm{and}\;\mathrm{Wet}/\mathrm{dry}\\ \mathrm{exergy}\;\mathrm{of}\;\mathrm{the}\;\mathrm{humid}\;\mathrm{air}\;\mathrm{containing}\;\mathrm{the}\;\mathrm{evaporated}\;\mathrm{water}\;\mathrm{from}\;\mathrm{the}\;\mathrm{sweat}]+\\ {}[\mathrm{Warm}/\mathrm{cool}\;\mathrm{radiant}\;\mathrm{exergy}\;\mathrm{discharged}\;\mathrm{from}\;\mathrm{the}\;\mathrm{whole}\;\mathrm{of}\;\mathrm{skin}\;\mathrm{and}\;\mathrm{clothing}\\ \mathrm{surfaces}]+[\mathrm{Warm}/\mathrm{cool}\;\mathrm{exergy}\;\mathrm{transferred}\;\mathrm{by}\;\mathrm{convection}\;\mathrm{from}\;\mathrm{the}\;\mathrm{whole}\;\mathrm{of}\;\mathrm{skin}\\ \mathrm{and}\;\mathrm{clothing}\;\mathrm{surfaces}\;\mathrm{into}\;\mathrm{the}\;\mathrm{surrounding}\;\mathrm{air}] \end{array}$$

The mathematical operations for the above equations are listed in the work by Shukuya et al. [26,27] and Shukuya [28]. The above used terms “warm” or “cool” exergy present the amount of exergy contained in a substance relative to its environment. The substance has “warm” exergy as a quantity of state if its temperature is higher than the environment, and “warm” exergy is the ability of thermal energy contained by the substance to disperse into the environment. The substance has “cool” exergy as a quantity of state if its temperature is lower than the environment, and “cool” exergy is the ability of the substance in which there is lack of thermal energy compared to the environment, to let the thermal energy in the environment flow into it [28]. In relation to human body exergy balance, warm radiant exergy absorbed by the whole of the skin and clothing surfaces appears in case of *T*_mr_ > *T*_ai_. Vice versa, *T*_mr_ < *T*_ai_ results in cool radiant exergy absorbed by the whole of skin and clothing surfaces. Warm radiant exergy discharged from the whole of skin and clothing surfaces appears in case of *T*_ai_ < *T*_cl_. Vice versa, *T*_ai_ > *T*_cl_ results in cool radiant exergy discharged from the whole of the skin and clothing surfaces. In case of convection, warm exergy transferred by convection from the whole of the skin and clothing surfaces into the surrounding air appears in case of *T*_ao_ < *T*_cl_ < *T*_ai_ and vice versa *T*_ai_ < *T*_cl_ < *T_ao_*, results in cool convective exergy discharged from the whole of the skin and clothing surfaces. Warm exergy transferred by convection from the whole of skin and clothing surfaces into the surrounding air appears in case of *T*_ao_ < *T*_ai_ < *T*_cl_ and *T*_ai_ < *T*_ao_ < *T*_cl_. Vice versa, *T*_cl_ < *T*_ao_ < *T*_ai_ and *T*_cl_ < *T*_ai_ < *T*_ao_, results in cool exergy transferred by convection from the whole of skin and clothing surfaces into the surrounding air (for our calculation we assume *T*_ao_ = *T*_ai_).

The first term of Equation (9) is warm exergy produced as the result of chemical exergy consumption for a variety of cellular activities, mainly for the contraction of muscle tissues, the composition of proteins and the sustenance of the relative concentrations of various minerals in the body cells. The metabolic exergy balance can be expressed as follows:

(10) $$\begin{array}{l} {}[\mathrm{Chemical}\;\mathrm{exergy}\;\mathrm{supply}]-[\mathrm{Exergy}\;\mathrm{consumption}]=[\mathrm{Exergy}\;\mathrm{supply}\;\mathrm{for}\;\mathrm{body}\\ \mathrm{function}]+[\mathrm{Warm}\;\mathrm{exergy}\;\mathrm{generated}] \end{array}$$

The last term in Equation (9) is exactly the warm exergy that appeared in the first term of Equation (9). The exergy consumption that appeared in the sixth term of Equation (9) is due to two kinds of dispersion: one is thermal dispersion caused by the temperature difference between the body core and the body shell (i.e., the skin, and the clothing surface); the rest is dispersion of liquid water into water vapour. In other words, free expansion of water molecules into the surrounding space. The chemical exergy consumption shown in Equation (10) usually amounts to more than 95% of chemical exergy supply. It implies that the amount of entropy generated in due course is very large, because the amount of entropy generated is exactly proportional to that of exergy consumption. All last five terms in Equation (9), except the exergy storage, play important roles in disposing of the generated entropy due to chemical exergy consumption within the human body, while at the same time disposing of the generated entropy due to thermal exergy consumption that appears in Equation (9). These processes of outgoing exergy flow and the exergy consumption influence significantly human well being: health and comfort [27] (p. 10).

The existing human body exergy model was modified as follows: *T*_sk_, *T*_cr_, *T*_cl_ were calculated according to input parameters for individual user and not for the average test subject; environmental parameters (*T*_o_, *T*_ai_, *T*_surf_, *T*_mr_) varied according to the tested system and presented real-time measurements. *T*_ai_ was assumed to be equal to *T*_ao_, *RH*_ai_ was assumed to be equal to *RH*_ao_. All parameters changed in time, and had an impact on the results of the balance. The main advantage of the modified model over the existing thermodynamic models is that it enables simultaneous treating of processes inside the individual human body and processes in the hospital room [28]. According to our research goals, we calculated inputs and outputs of the human body exergy balance as well as the human body exergy consumption (hBExC) rate valid for thermoregulation (i.e., destroyed exergy rate) using spreadsheet software by Hideo Asada Rev 2010 [26,28].

To maintain homeostatic conditions, it is important that the optimal hbExC rate and stored exergy values are attained with an efficient combination of exergy input and exergy output. The optimal values of hbExC, stored exergy, exergy inputs and outputs are mainly influenced by the characteristics of individual users. In order to achieve healing-oriented conditions for burn patients, our goal is first to create conditions that will result in minimal possible use of hbExC, besides minimal radiation, convection and evaporation. Second, for other users, thermally comfortable conditions equal to thermal neutrality shall be achieved, besides the lower hbExC rate valid for thermoregulation. Thermal neutrality is the energy-wise condition, where internal heat production is equal to the sum of heat losses from the body (i.e., neutral thermal load on the human body, *L* = 0).

## 3. Results

According to objectives of our study, the results are divided into the following sub-sections: design morphology of healing environment; a set of influencing parameters for hospital design; definition of individual needs, demands and characteristics for a burn patient room; and development and testing of the innovative UCCPS.

### 3.1. Design Morphology of Healing Environment

**Figure 2:**
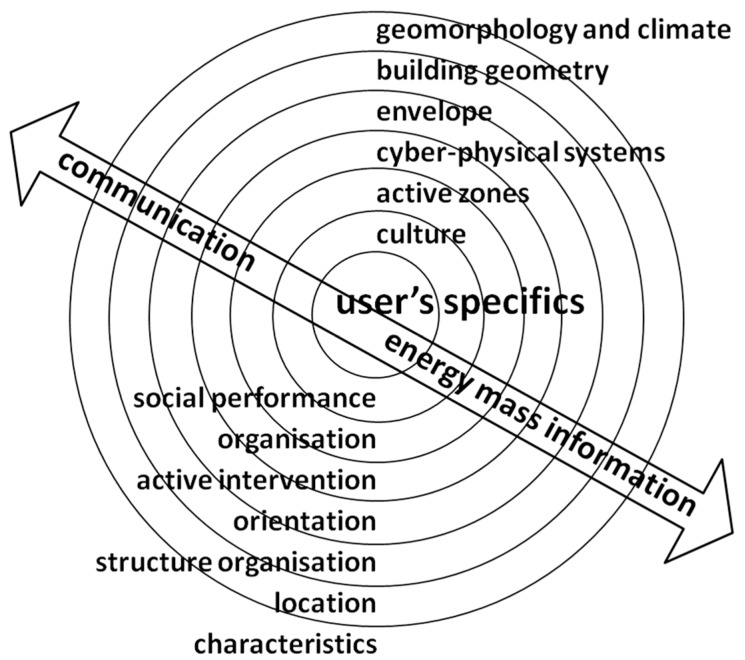
Model of User Building System.

Design morphology of a healing environment has to follow the basic principles of bioclimatic design (Figure 2). The starting point is the purpose of the building, the design of which follows the location endorsement [29,30] and interactivity of influences in smart house design [31]. Transparent and non-transparent parts of the building envelope present the interface between the exterior and interior built environment. Their flexibility enables maximal exploitation of renewable energy resources and protection against negative impacts (i.e., heat, light, mass transfer). The centre of our design is individual users, in our case vulnerable patients, with specific needs and demands. Definition of the needs and demands is the guidance for the design of active zones (i.e., patient and work rooms) with selection of efficient cyber-physical systems.

A cyber-physical system consists of three subcomponents: 1. active, multifunctional building envelope; 2. LowEx devices, and 3. control and information system. The LowEx system includes radiant surfaces in the room with changeable positions: walls, ceiling, floor (final area). It is connected with a control and information system that subordinates it to individualized user requirements in the room. It is important to stress that the heating-cooling system works independently from the regulation of ventilation and humidification. It includes local 3rd generation distribution and heat emission: heating and cooling; a fresh air supply with appropriate filters; and discharge of contaminated air from the room. Only such an integral design approach enables the implementation of a healing environment paradigm.

### 3.2. A Set of Influencing Parameters for Hospital Design

IEQ conditions, in the context of a healing environment depend on the characteristics of the building envelope (composition of transparent and non-transparent parts with building-physical characteristics) and the characteristics of the users and cyber-physical system. Designers of healing environment must be fully acquainted with all IEQ influential factors and their interrelations, such as thermal comfort, visual comfort and non-image forming effects, air quality, acoustics, ergonomics, together with the principles of engineering design [32]. Knowing and understanding influential factors and their interrelations is crucial for every design stage, where many negative influences that deteriorate IEQ, can be promptly prevented and controlled. With this concept, a matrix of influencing parameters related to IEQ, building and systems was designed and presented in Table 1. The matrix is a tool, applicable for every step and level of hospital design, i.e., the selection of location, the design of the building envelope, the selection of materials for constructional complexes, the type of HVAC systems, etc. The presented matrix includes relevant parameters related to building physics (e.g., heat capacity of material, thermal transmittance of constructional complexes, light transmittance of glass, etc.) and parameters related to IEQ (e.g., indoor relative humidity, indoor air temperature, etc.). It is an open matrix, where parameters can be changed or added, according to the stage or focus of our design. Besides parameters, the interactions between them are identified, according to the relevant literature search (evidence based approach). Systematically overviewed parameters and their interactions enable holistic actions. Namely, if the designer changes one building parameter (e.g., the decrease of overall heat capacity of the walls), this might affect other IEQ parameters (e.g., the increase of interior wall surface temperature during cooling season that leads to uncomfortable air and operative temperatures). In order to create optimal thermal conditions, air and operative temperatures have to be decreased actively by the installed systems (note: currently solved by AC systems; the problem presented in introduction). Our approach enables us to define healthier and more sustainable step-by-step measures that result in optimal IEQ as well as lower energy use (e.g., efficient building envelope and HVAC systems, optimal surface temperatures). All identified interactions are marked by their influential level (i.e., very high, high, medium, low, very low influential level and not detected influential level) that can be used for defining preferable actions or those applicable in the design of smart control systems based on fuzzy logic.

### 3.3. Definition of Specific User Needs and Demands

In a hospital environment, of key importance is to attain the optimal conditions for healthcare and medical treatment as well as comfortable conditions for other users. Optimal conditions depend on the user’s characteristics.

Table 2 presents an example of identified individual characteristics (e.g., *T*_sk_, *T*_cl_, Met) for three users of burn patient room: patient, healthcare worker and visitor. It was prepared on the basis of literature review, as a part of the evidence-based design. Additionally, it can be completed according to special design requirements, including influential level ranking, measured values, etc.

Individual needs, demands and characteristics are directly related to the selection and creation of optimal conditions for individual users. The required optimal conditions present the required and recommended values for active spaces and depend on the building and its systems. In our case, the required and recommended indoor environmental conditions for a burn patient room equipped with conventional system or UCCPS are:

- Requirements for burn patient zone crated by conventional system. A ward for severe burn injuries should have temperature controls that permit adjusting the room temperature up to 32 °C and relative humidity up to 95% [9]. *T*_ai_ and *RH*_ai_ should be maintained at 30–33 °C and 80%, respectively, in order to decrease energy demands and evaporative heat losses [23,24].
- Requirements for zone for a visitor and healthcare worker created by conventional system. The required air temperatures range from 20 °C to 26 °C due to the specifics of the ward facility [9,33,34] recommends maintaining the relative humidity in occupied spaces in the range from 30% to 60% and air temperature between 20 °C and 25 °C.
- Requirements for healing and comfort conditions created by UCCPS. Regulations and recommendations for hospital environment define requirements for *T*_ai_ and *RH*_ai_ that are useful for a room equipped with the conventional system. For the room with the UCCPS, *T*_o_ was introduced and presents together with 80% *RH*_ai_ the required condition (*T*_o_ = 32 °C, *RH*_ai_ = 80%) [35]. *T*_o_ was created as a combination between mean radiant temperature *T*_mr_ and *T*_ai_.

All identified individual characteristics as well as the required and/or recommend indoor environmental conditions were used for exergy calculations (design and testing of the UCCPS), presented in the following section.

### 3.4. Why Is the Creation of the Required Environmental Conditions Important for Burn Patients?

A burn patient is from thermodynamic point of view a very specific subject. A burn patient has, regarding medical data [9,23,24,36,37,38,43,52,53,54,55,56,57,58,59,60,61,62,63,64,65,66,67], higher *T*_sk_, lower *T*_cr_ and higher metabolic rate than a healthy individual. Such specific user characteristics require hot and humid environmental conditions. Due to the fact that such conditions are in current design practice often not fulfilled, their influence was considered from a thermodynamic point of view and presented in the following section.

**Figure 3:**
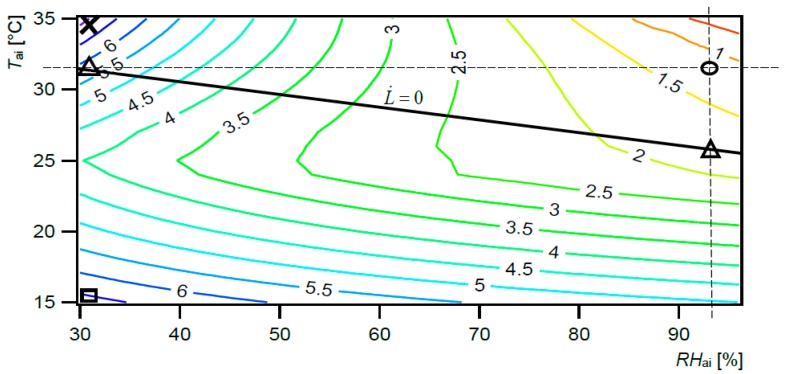
Exergy consumption rate for burn patient, human body exergy consumption (hbExC) rate [W/m^2^], as a function of *T*_ai_ (=*T*_mr_) [°C] and *RH*_ai_ [%]. Black line: rate of thermal load on the body surface area ($\dot{L}$ = 0).

Figure 3 presents the hbExC rate valid for the thermoregulation of burn patient as a function of *T*_ai_ (=*T*_mr_) and *RH*_ai_. The hbExC rate of burn patient varies from 0.17–7.17 W/m^2^, depending on the environmental conditions. According to Equations (1)–(9), the calculated value of the hbExC rate (destroyed exergy) for a burn patient is mainly affected by input metabolic thermal exergy rate and output exergy rates by radiation, convection, exhalation and evaporation of sweat.

The hbExC rate is the lowest possible rate in the required conditions (circle mark: 0.17 W/m^2^; 32 °C, 95%), mainly due to minimum rate of input exergy by metabolic thermal exergy rate. If we expose a burn patient to conditions different than those required, for example a cold and dry environment, the rate of exergy consumption will become much higher due to higher exergy consumption rate (square mark: 6.7 W/m^2^; 15 °C, 30%).

The worst case is high *T*_ai_ and low *RH*_ai_, where the exergy consumption rate is the highest (X mark: 7.17 W/m^2^; 35 °C, 30%), mainly due to high input exergy by metabolic thermal exergy rate. Although the air temperature in the range from 28 °C–32 °C results in $\dot{L}=0$ (i.e., internal heat production is equal to the sum of heat losses from the body), the hbExC rate is not the minimal possible in these conditions (it ranges from 1.7 to 5.8 W/m^2^, triangle mark). Only the required conditions with high *T*_ai_ 32 °C, and low *RH*_ai_ 95% result in the lowest possible hbExC rate valid for thermoregulation (0.17 W/m^2^). Therefore, designers must realize that it is important to attain both the required *T*_ai_ and *RH*_ai_ in combination.

The thermodynamic response of a burn patient depends not only on the environmental conditions, but also on the type of the installed heating and cooling system. Because of this, conventional and user-centred cyber-physical heating and cooling systems were compared from the aspect of thermodynamic response of a burn patient.

### 3.5. Has the Selected Type of the Heating and Cooling System Impacted on the Thermodynamic Response of Burn Patients?

The required conditions for the test room were created separately with conventional system (*T*_ai_ = *T*_mr_ = *T*_o_ 32 °C, *RH*_ai_ 80%) and with UCCPS *(T*_ai_ ≠ *T*_mr_, *T*_o_ 32 °C, *RH*_ai_ 80%). To allow comparison between the conventional system and UCCPS, the whole human body exergy balance is taken into consideration. Table 3 shows the results of calculated values of exergy inputs, exergy outputs, hbExC and stored exergy for burn patient exposed to conditions created with the conventional system (*T*_ai_ = *T*_mr_ = 32 °C) and UCCPS (*T*_ai_ = 35 °C, *T*_mr_ = 31 °C).

In the required environmental conditions created by a conventional system, a burn patient has: (1) higher hbExC rate valid for thermoregulation (1.79 W/m^2^); (2) higher rates of exergy inputs by metabolic thermal exergy rate (2.29 W/m^2^); (3) higher rate of stored exergy (0.006 W/m^2^); (4) higher rates of exergy outputs by warm radiant and convective exergy transferred from the whole skin and clothing surfaces (0.12 W/m^2^, 0.32 W/m^2^); and (5) higher by exhalation and evaporation of sweat (0.05 W/m^2^), compared to the conditions created by the UCCPS.

Required environmental conditions created by the UCCPS result in: (1) lower values of the hbExC rate (1.36 W/m^2^); (2) lower rates of input exergy by metabolic thermal exergy (1.30 W/m^2^); and (3) cool radiant exergy absorbed from the whole skin and clothing surfaces (0.07 W/m^2^); (4) lower rate of stored exergy (0.001 W/m^2^); and (5) lower rates of output exergy by warm radiation and warm convection transferred from the whole skin and clothing surfaces (0.01 W/m^2^, 0.03 W/m^2^) than in conditions created by a conventional system.

As presented, the type of installed system has an important influence on separate parts of the human body thermal balance.

### 3.6. Is It Possible to Actively Regulate Conditions in a Healing Environment by Taking into Consideration the Needs, Demands and Characteristics of Individual Users?

With the goal to achieve optimal environmental conditions for health care and treatment inside the healing environment, the installed system shall regulate separate environmental parameters according to the user specifics. However, current conventional systems only enable the regulation of *T*_ai_ (as a set temperature, *T*_set up_: *T*_ai_ = *T*_mr_ = *T*_o_ 32 °C). This results in unchangeable values of hBExC rate (1.79 W/m^2^) and human body exergy balance (Table 3). Inputs and outputs of the human body exergy balance (i.e., radiation, convection, exhalation and evaporation of sweat), cannot be regulated by this system.

By contrast with the conventional system, UCCPS enables the setting of various *T*_set up_ according to the required *T*_o_, namely by setting combinations between *T*_ai_ and *T*_mr_. Table 4 summarizes the values of the exergy balances for burn patient exposed to conditions created with the UCCPS. The system gives us new possibilities to control and regulate every part of the human body exergy balance according to the user specifics. In such a way, possibilities of active regulation of healing, health and comfort conditions for individual user are revealed. The condition to be taken as a priority depends considerably on the regulated demands and individual needs.

**Figure 4:**
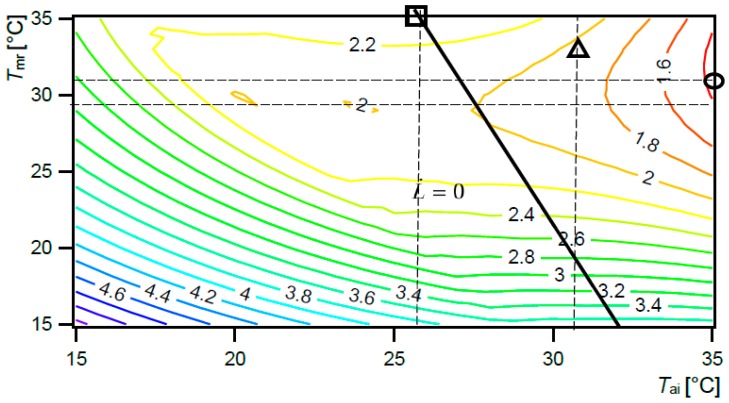
Exergy consumption (hbExC) rate for burn patient [W/m^2^], as a function of *T*_ai_ and *T*_mr_, 80% *RH*_ai_, UCCPS. Black line: rate of thermal load on the body surface area being zero ($\dot{L}$ = 0).

The set-up temperatures for the system depend on the priority requirements and conditions for individual user: healing or comfort. For example, for burn patients we have to create healing oriented conditions with minimal possible hbExC rate, besides minimal exhalation and evaporation of sweat, radiation and convection. Although the recommended value *T*_o_ 32 °C can be fulfilled with a combination of *T*_mr_ in a range 31–35 °C, and *T*_ai_ in a range 26–35 °C, we have to select such a *T*_set up_ that results in a minimal possible human body exergy consumption rate in burn patient, which is 31 °C *T*_mr_ and 35 °C *T*_ai_ (circle mark) (Figure 4). In this way, optimal healing conditions (recommended *T*_o_ 32 °C) are created with a minimal human body exergy consumption rate (1.36 W/m^2^) and minimal exergy rate of exhalation and evaporation of sweat (0.02 W/m^2^), warm radiant exergy rate (0.01 W/m^2^) and warm convective exergy rate transferred from the whole skin and clothing surfaces (0.03 W/m^2^) (Table 4). On the other hand, comfort conditions with $\dot{L}$ = 0 (black line) do not result in a minimal possible hbExC rate and minimal evaporation, radiation and convection (as presented in the previous section). For example, conditions with *T*_ai_ 26 °C and *T*_mr_ 35 °C are required conditions with *T*_o_ 32 °C and comfort conditions $\dot{L}$ = 0 as well, but they result in a higher exergy consumption rate of 2.37 W/m^2^ (square mark, Figure 4), and higher losses by exergy rate of exhalation and evaporation of sweat 0.16 W/m^2^, warm radiant exergy rate 0.67 W/m^2^ and warm convective exergy rate transferred from the whole skin and clothing surfaces 1.80 W/m^2^ (Table 4). All the values of the balance are higher than they were in conditions with *T*_mr_ to 31 °C and *T*_ai_ to 35 °C.

**Figure 5:**
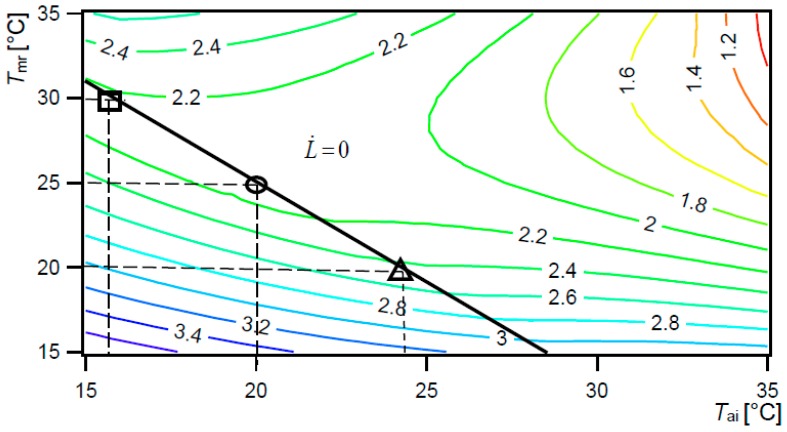
Exergy consumption (hbExC) rate for healthcare worker [W/m^2^], as a function of *T*_ai_ and *T*_mr_, 80% *RH*_ai_, user-centred cyber-physical system. Black line: rate of thermal load on the body surface area being zero ($\dot{L}$ = 0).

Similar to active regulation of healing conditions for burn patients, comfort conditions for other users can also be regulated. These conditions result in $\dot{L}=0$ and minimal possible human body exergy consumption rate (black line, Figure 5). Therefore, a UCCPS for healthcare worker could be set up to the conditions *T*_ai_ 16 °C and *T*_mr_ 30 °C (*T*_o_ 25.6 °C) with exergy consumption rate 2.20 W/m^2^ (square mark), or *T*_ai_ 24 °C and *T*_mr_ 20 °C (*T*_o_ 21.3 °C) with an exergy consumption rate of 2.43 W/m^2^ (triangle mark), and *T*_ai_ 20 °C and *T*_mr_ 25 °C (*T*_o_ 23.4 °C) with an exergy consumption rate of 2.10 W/m^2^ (cycle mark). All these conditions result in $\dot{L}$ = 0. If the minimal possible exergy consumption rate (2.10 W/m^2^) and comfort conditions ($\dot{L}$ = 0) are to be attained at the same time, *T*_ai_ and *T*_mr_ have to be 20 °C and 25 °C (*T*_o_ = 23.4 °C), respectively.

**Figure 6:**
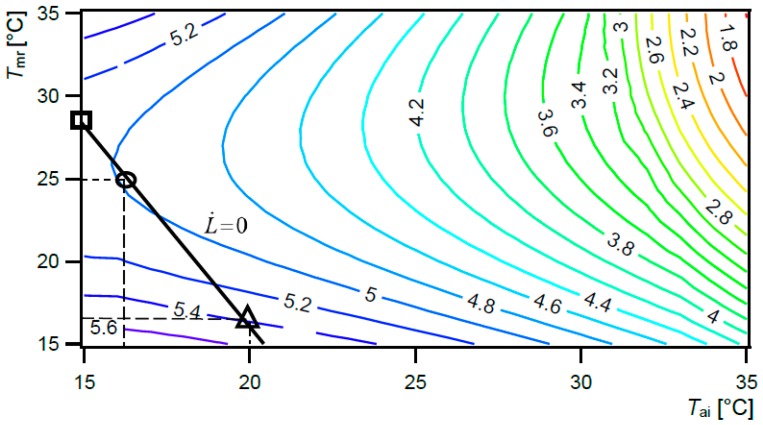
Exergy consumption (hbExC) rate for visitor [W/m^2^], *T*_ai_ (*T*_mr_)*:* UCCPS. Black line: rate of thermal load on the body surface area being zero ($\dot{L}$ = 0).

The UCCPS for a visitor could be set up to *T*_ai_ 17.0 °C, *T*_mr_ 25 °C (*T*_o_ = 22.3 °C) with 4.95 W/m^2^ of human body exergy consumption rate (cycle mark, Figure 6) or *T*_ai_ 15 °C and *T*_mr_ 29 °C with an exergy consumption rate of 5.1 W/m^2^ (square mark) or *T*_ai_ 16 °C and *T*_mr_ 20 °C with an exergy consumption rate of 5.5 W/m^2^ (triangle mark). If minimal possible exergy consumption rate (2.10 W/m^2^) and comfort conditions ($\dot{L}$ = 0) are to be attained at the same time, *T*_ai_ and *T*_mr_ have to be 17 °C and 25 °C (*T*_o_ = 22.3 °C), respectively. Thus, optimal conditions for a visitor are created, with a minimal possible human body exergy consumption rate and attained comfort conditions ($\dot{L}$ = 0).

As presented, the designed system actively regulates the conditions in healing environment by considering the needs, demands and characteristics of individual users.

## 4. Discussion

Until now, many studies have proven the positive effects of a healing environment on patients, staff and visitors [15,17,18,19,20,22]. However, there is no developed integral methodology for the design of a healing environment that is based on simultaneous treatment of users, building systems and place dynamic interactions. Additionally, there is no designed system that enables the attainment of a healing environment for individual users of an active space. Our paper presents the process of design of a healing environment with a tested system that enables the attainment not only of healthy and comfortable conditions, but also of healing-oriented conditions for individual users. Placing the user in front of the design is an important and indispensable step for future extensive renovations. At present design and construction health is frequently the victim of energetic and economic issues. The required conditions need to be created especially in hospital buildings, where the probability of adverse health effects of inadequate environment on users is rising.

Creating healing-oriented conditions is important for a burn patient, who from a thermodynamic point of view is a very specific subject. Thermal injury results in significant pathophysiological changes that start with an initial critical phase, continue with an intermediate phase, and end with a second critical phase. During the intermediate phase, there appear metabolic changes that lead to increased metabolic rates, and higher or lower body core temperature due to a reset thermostatic control centre in the hypothalamus [23] (p. 69), [37,38]. Hormonal (i.e., secretion of stress hormones and metabolic mediators) and metabolic responses (i.e., increase in resting energy expenditure, heart rate, blood pressure, minute ventilation) to a burn injury lead to hypermetabolism, progressive weight loss, increased susceptibility to infection, and poor wound healing [24,39,41,43,52,59,66]. Burn patients are by far the most susceptible to intr- and post-operative hypothermia, since the damaged skin is no longer able to prevent the loss of body heat [37]. Patients with extensive thermal injuries have a tremendous, long-lasting increase in transcutaneous heat loss by increased radiation, convection and evaporation [24,41,43,52,53,54,55,58,66]. To minimize the hypermetabolic response of a burn patient, environmental warming is one of the most important actions [23,24] (p. 237); the increased ambient temperature attenuates hypermetabolism and increases patient comfort.

A system that enables the attainment of a healing environment for individual users of active space was designed by the exergy concept that treats the process in the individual human body in relation to environmental conditions in the patient room. The exergy concept has been introduced in various disciplines with the goal to optimize natural and human systems. The use of the exergy concept in the built environment was first introduced in the 1990s. Since the 2000s, studies have been focused on the human body exergy balance in relation to various environmental conditions [28,68,69,70,71,72,73,74]. Currently, much effort has been put in the development of unsteady-state human-body exergy analyses in relation to dynamic thermal environments [75]. Additionally, the performance indicators for individuals under physical activity based on the concepts of exergy destroyed and exergy efficiency were applied in the study by Mady et al. [76]. An exergy model of the human heart for normotensive and hypertensive people was developed by Henriques et al. [77].

The exergy analysis performed proved that the hbExC rate for burn patient is the lowest possible in hot and humid environment (32 °C, 95%), which is in the required conditions. The same conclusions were made by Herndon [60] and Wilmore et al. [24]. Wilmore et al. [24] found out that hypermetabolism can be attenuated by increasing the ambient temperature from 25 to 33 °C. Herndon [60] concluded that metabolic rate is the lowest possible in an ambient temperature of 35 °C. In these two studies, only *T*_ai_ was taken into consideration, but not *RH*_ai_. Our study reveals the importance of creating high *T*_ai_ together with high *RH*_ai_. From a thermodynamic point of view, the worst condition is high *T*_ai_ and low *RH*_ai_, where the hbExC rate is the highest, mainly due to increased input exergy rate by the value of metabolic thermal exergy rate.

The selected type of heating and cooling system has an impact on thermodynamic response of a burn patient. The comparison between the two systems shows that a more optimal human body exergy balance with lower values of hbExC rate, evaporation, convection and radiation is attained by UCCPS. Moreover, the possibility of active regulation of healing conditions by taking into consideration the needs and demands of individual users is revealed.

The approach presented is important because there are always individual differences in the perception of a thermal environment in the built environment. Therefore, in hospitals, the heating and cooling system shall distinguish between the required conditions for patients and comfort conditions for staff and visitors. The importance of finding acceptable solutions for various thermal comfort requirements was highlighted by Khodakarami and Nasrollahi [78]. By simultaneous analysis of the thermal process inside an individual human body and a thermal process in an active space, the exact parameters for the functioning of a user-centred system was defined. Therefore, UCCPS enables the creation of optimal conditions for the user individually, by setting up an optimal relation between *T*_ai_ and *T*_mr_. In hospitals, it is up to medical staff to decide what kind of combination of influential factors, such as *T*_ai_, *T*_mr_, *RH*_ai_ etc., will be included in the regulation of separate parts of the human body exergy balance and to take into account other specific factors using their medical expertise.

In such a way, our system actively regulates healing-oriented conditions based on the target impacts of an internal environment, which are conditions important for health care and treatment. Based on the exergetic analysis of thermodynamic response of burn patient, set up parameters are *T*_ai_ 35.0 °C and *T*_mr_ 31.0 °C (*T*_o_ 32.0 °C). In the case of a burn patient, the required conditions cause the lowest possible internal production by metabolism and also the lowest evaporation, besides low convection and radiation, as mentioned by Herndon [60] and Herndon [23] (p. 492). These required conditions are healing-oriented conditions for a patient and cause the lowest possible human body exergy consumption rate valid for thermoregulation, lower metabolic thermal exergy rate, and also lower exergy rates of exhalation and evaporation of sweat, radiation and convection, as mentioned by Caldwell [53,54], Caldwell et al. [55], Cone at al. [58], Herndon et al. [61,62], Herndon and Parks [63], Herndon and Tompkins [64], Kelemen et al. [65], Marin et al. [43], Wallace et al. [66], and Wilmore et al. [24]. If we expose a burn patient to conditions that differ from the required ones, the exergy consumption rate will become higher, due to higher metabolic thermal exergy rate, together with higher exergy rates of evaporation and exhalation of sweat, radiation and convection.

**Figure 7:**
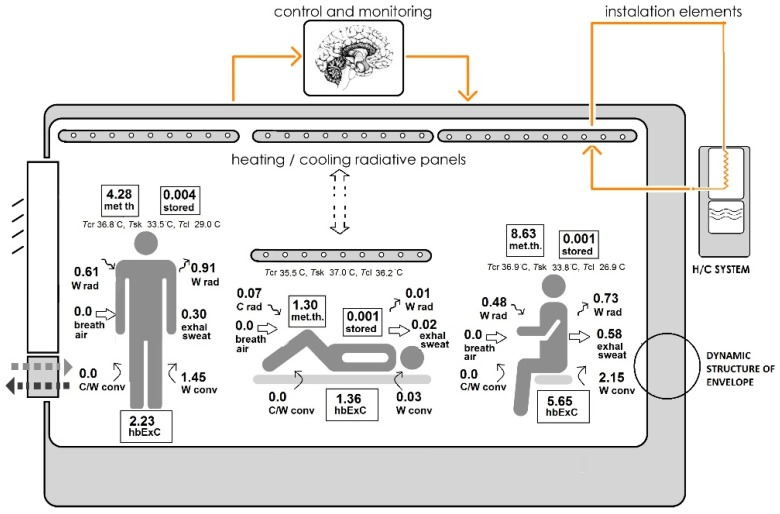
Active regulation of healing oriented conditions and thermally comfortable conditions by UCCPS: (a) healthcare worker (*T*_ai_ 18.0 °C, *T*_mr_ 27.0 °C, *T*_o_ 24.1 °C, *RH*_ai_ 60%); (b) burn patient (*T*_ai_ 35.0 °C, *T*_mr_ 31.0 °C, *T*_o_ 32.0 °C, *RH*_ai_ 80%); (c) visitor (*T*_ai_ 17.0 °C, *T*_mr_ 25.0 °C, *T*_o_ 22.3 °C, *RH*_ai_ 60%). Abbreviations: breath air, sum of exergies contained by the inhaled humid air; C/W conv in/out, warm/cool convective exergy absorbed by/discharged from the whole skin and clothing surfaces into the surrounding air; C/W rad in/out, warm/cool radiant exergy absorbed by/discharged from the whole skin and clothing surfaces; exhal sweat, exhalation and evaporation of sweat; H/C system, heating and cooling system; hbExC, human body exergy consumption rate valid for thermoregulation; met th, metabolic thermal exergy; stored, stored exergy in the core and in the shell; *T*_ai_, room air temperature; *T*_cl_, clothing temperature; *T*_cr_, body core temperature; *T*_mr_, mean radiant temperature; *T*_o_, operative temperature; *T*_sk_, skin temperature; *RH*_ai_, relative humidity of indoor air.

The main advantage of the designed system is its flexibility. As presented in Figure 7, the system enables zoning of space, namely the creation of an active zone inside the active space, with no deterioration of the required conditions for a burn patient. The system creates optimal environmental conditions for other users of an active space by setting up *T*_set up_, i.e., *T*_ai_ 18.0 °C, *T*_mr_ 27.0 °C, (*T*_o_ 24.1 °C) and 60% *RH*_ai_ for a health care worker and *T*_ai_ 17.0 °C, *T*_mr_ 25.0 °C, (*T*_o_ 22.3 °C) and 60% *RH*_ai_ for a visitor. This results in thermal comfort conditions with minimal possible hbExC rate valid for thermoregulation (2.23 W/m^2^ for health care worker and 5.65 W/m^2^ for visitor). The association between comfortable conditions and lower hbExC rate was proven by Shukuya et al. [26,27,28] and Henriques [79]: “Thermal neutrality leads to lower human body exergy consumption (hbExC) rate valid for thermoregulation.” [26,27,28,79].

To provide optimal environmental conditions for individual users it is necessary to pay attention not only to efficient heating and cooling system, but also to the application of dynamic building envelope with controlled surface temperature (matrix, Table 1). It is also important to take into consideration the thermal capacity of building envelope parts [80,81] as well as environmental issues [82,83]. During a heating season, poorly insulated building envelope and the use of a conventional system cause lower surface temperatures. Lower surface temperatures result in lower rate of warm radiant exergy absorbed by the whole skin and clothing surfaces and also lower rates of warm exergies by radiation and convection discharged from the whole skin and clothing surfaces. Vice versa, during cooling season, higher surface temperatures result in lower cool radiant exergy absorbed by the whole skin and clothing surfaces and also lower rates of cool exergies by radiation and convection discharged from the whole skin and clothing surfaces. Moreover, another problem is also air-conditioning often used in the majority of hospital buildings. Air-conditioning units extinguish or kill the “warm” exergy flow into the room space very brutally by large consumption of “cool” exergy. All these conditions lead to discomfort and have to be avoided.

A correctly structured building envelope within UCCPS importantly benefits separate values of the human body exergy balance in all seasons; it decreases the hbExC rate due to lower input exergy and higher output exergy. Such an integral design results not only in improved IEQ conditions, but also to significant energy savings. Hospitals are the largest non-residential buildings that consume 10% of final energy considering all non-residential buildings in the EU. Specific energy use in an EU hospital is approximately 280–430 kWh/(m^2^a) [8]. Studies show that the installation of low temperature heating and a high temperature cooling system resulted in up to 27% lower measured energy use for space heating and up to 73% lower measured energy use for space cooling compared to the conventional system [35]. An integral approach to interventions performed on building envelope systems with efficient heating and cooling systems resulted in 57% of total building exergy saving potential [35].

### Strengths and Limitations

The presented study reveals the main difficulties in the existing design practice of hospitals that are reflected in unhealthy, uncomfortable and unproductive conditions. Information on these aid in the renovation and construction process of all other living and working environments used by vulnerable population groups.

The study presents the first example of design morphology of a healing environment and a system that enables the attainment of healing-oriented conditions for individual users. Our morphology can be implemented in the existing decision models for sustainable hospital building construction and renovation [84]. The design was performed on the basis of the exergy analyses that simultaneously treat processes inside the individual human body and processes in the burn patient room. An extremely demanding environment with specific subject characteristics was chosen in order for this to be easily transferred to any other environment and used for any other user with different and/or less demanding environments. Thermodynamic response was calculated for individual users based on the modification of the existing exergy model. The designed system was tested in a real test environment and on a model scale. Human body exergy balance was calculated for various environmental conditions. In previous studies, only *T*_ai_ was taken into consideration, but not *RH*_ai_. An innovation of the designed UCCPS is the active regulation of healing and comfort conditions according to the needs, demands and characteristics of individual users. Healing conditions are not necessarily the same as comfort conditions. This aspect is very important and gives us totally new possibilities in curative or preventive medicine.

The main limitation of our study is that only 9 m^2^ of celling was covered by panels. The thermodynamic response was analysed on the basis of virtual users. In further work, it should be beneficial to analyse the effect of changing the surface temperatures of the whole room envelope. Together with these, other parameters of IEQ should also be actively regulated by our proposed system. For example, in the case of efficient ventilation systems, personalised ventilation systems [85], and local systems are highly applicable. A selected type of the system has to fulfil all the requirements from the sanitary-technical and hygienic point of view (e.g., maintenance, use of healthy materials, etc.). Finally, the required ventilation levels have to be optimal and not physiological minimal values, as was presented in the introduction. The most important advantage of the system presented here is in the healing/cooling radiative panels, where the thermal part functions independently of the ventilation part. This is not in the case for existing air-conditioning systems, where the same air is used as an energy carrier and for ventilation.

## 5. Conclusions

The role of health in the process of building design is becoming increasingly important, which is also evident from the new EU legislation, Energy Performance of Buildings Directive (EPBD) proposal [86]: “Better performing buildings provide higher comfort levels and wellbeing for their occupants and improve health by reducing mortality and morbidity from a poor indoor climate. Adequately heated and ventilated dwellings alleviate negative health impacts caused by dampness, particularly amongst vulnerable groups such as children and the elderly and those with pre-existing illnesses” [86] (p. 2).

Interventions presented in this paper are a guidance for future extensive renovations and the construction of hospitals. User-centred design with an emphasis on vulnerable population groups has an important role in the environmental health activities:

- Each action towards building energy efficiency has to take into account the complexity of the reciprocal interaction between the treated environment and the user, and it has to be not only directed towards healthy and comfortable conditions, but also towards healing environments supported by energy efficient systems.
- Any deviation of environmental conditions from the required values results in deteriorated patient treatment outcome, uncomfortable conditions and decreased productivity. This is especially important for burn patients, where mortality, morbidity and hospitalization can be significantly decreased with optimal environmental conditions. Therefore, in burn patient rooms every deviation from the required values results in a higher human body exergy consumption rate valid for thermoregulation, besides higher metabolic thermal exergy rate and also higher exergy rates of exhalation and evaporation of sweat, radiation and convection. In order to improve patient treatment outcomes, these conditions have to be prevented.
- An innovative user-centred cyber-physical system (UCCPS) in a healing environment enables us to attain optimal thermal balance that may be individually regulated according to the user. Set up system parameters for burn patients result in the lowest possible human body exergy consumption rate valid for thermoregulation, a lower metabolic thermal exergy rate and also lower exergy rates of exhalation and evaporation of sweat, radiation and convection. Set up system parameters for healthcare workers and visitors result in thermally comfortable conditions with minimal hbExC rate and neutral thermal load on their body.
- The thermodynamic response of the human body is influenced not only by the installed heating and cooling system but also by the characteristics of the building envelope. Therefore, it is necessary to create a multi-levelled and dynamic process for the design of a healing environment. A hospital design that includes a dynamic building envelope with efficient systems results not only in improved indoor environmental conditions but also in a significant reduction of energy use.
- Information on this is an aid in the design of any other living and working environment with vulnerable population groups.

## Figures and Tables

**Table 1:** Matrix of influencing parameters for hospital design.

Building and System Components	Parameter		Building Envelope										System			
			Non-Transparent						Transparent				Heating/Cooling		Ventilation	Air Conditioning
			1	2	3	4	5	6	7	8	9	10	11	12	13	14
IEQ Issues			*U*_nt_ [W/(m^2^K)]	*C* [kJ/(kgK)]	*ν* [-]	*η* [h]	*X*_diff_ [%]	*Air Tightness* [h^−1^]	*U*_tr_ [W/(m^2^K)]]	*g* [-]	*τ*_ao_ [-]	*SHGC* [-]	*T*_medium_ [°C]	*T*_surf_ [°C]	Ventilation Rate [m^3^/h per Person]	*RH*_ai_ [%]
Thermal Comfort	1	*T*_ai_ [°C]	1/1 H	1/2 H	1/3 H	1/4 H	1/5 VL	1/6 VL	1/7 H	-	-	1/10 H	1/11 H	1/12 VH	-	1/14 H
	2	*T*_mr_ [°C]	2/1 VH	2/2 VH	2/3VH	2/4 VH	2/5 VL	2/6 VL	2/7 VH		-	2/10 VH	2/11 VH	2/12 VH	-	2/14 M
	3	*T*_o_ [°C]	3/1 VH	3/2 VH	3/3 VH	3/4 VH	3/5 VL	3/6 VL	3/7 VH	-	-	3/10 VH	3/11 VH	3/12 VH	-	3/14 M
	4	*RH*_ai_ [°C]	4/1 VL	4/2 VL	4/3 VL	4/4 VL	4/5 M	4/6 VL	4/7 VL	-	-	4/10nVL	4/11 H	4/12 L	-	4/14 VH
	5	*v*_ai_ [°C]	5/1 VL	5/2 VL	5/3 VL	5/4 VL	5/5 VL	5/6 H	5/7 VL	-	-	5/10 VL	5/11 VL	5/12 VL	5/13 VH	5/14 VL
	6	Met [met]	6/1 VL	6/2 VL	6/3 VL	6/4 VL	6/5 VL	6/6 VL	6/7 VL	-	-	6/10 VL	6/11 VL	6/12 VL	-	6/14 VL
	7	Clo [clo]	7/1 L	7/2 L	7/3 VL	7/4 VL	7/5 VL	7/6 VL	7/7 VL	-	-	7/10 VL	7/11 VL	7/12 M	-	7/14 M
Indoor Air Quality	8	Bioeffluents, Bioaerosols (e.q. CO_2_) [mg/m^3^, ppm]	8/1 VL	8/2 VL	-	-	8/5 VL	8/6 M	8/7 VL	-	-	8/10 VL	8/11 VL	8/12 VL	8/13 VH	8/14 L
	9	Hidden Olfs, Emission from Construction Products (e.g., VOCs, CH_2_O, phthalate esters) [mg/m^3^, ppm]	9/1 VL	9/2 VL	-	-	9/5 H	9/6 M	9/7 VL	-	-	9/10 VL	9/11 M	9/12 H	9/13 H	9/14 H
	10	Radon [Bq/m^3^]	10/1 VL	10/2 VL	-	-	10/5 VL	10/6 VH	10/7 VL	-	-	10/10 VL	10/11 VL	10/12 VL	10/13 VH	10/14 VL
	11	Bacteria, Moulds, Viruses	11/1 M	11/2 M	-	-	11/5 VH	11/6 VL	11/7 M	11/8 M	11/9 M	11/10 M	11/11 M	11/12 H	11/13 VH	11/14 VH
Visual Comfort and Non-Image Forming Effects	12	*E*_h_, *E*_v_ [lx]	-	-		-	-	-	12/7 VH	12/8 VH	12/9 VH	12/10 VH	-	-	-	-
	13	*DF* [%]	-	-	-	-	-	-	13/7 VH	13/8 VH	13/9 VH	13/10 VH	-	-	-	-
	14	*T*_CP_ [K]	-	-	-	-	-	-	14/7 VH	14/8 VH	14/9 VH	14/10 VH	-	-	-	-
	15	*U*_o_ [-]	-	-	-	-	-	-	15/7 VH	15/8 VH	15/9 VH	15/10 VH	-	-	-	-
	16	*DGP* [-]	-	-	-	-	-	-	16/7 VH	16/8 VH	16/9 VH	16/10 VH	-	-	-	-
	17	Wavelength, Time Availability, Spatial Distribution, CS, MS	-	-	-	-	-	-	17/7 VH	17/8 VH	17/9 VH	17/10 VH	-	-	-	-
Room Acoustic Noise, Vibrations	18	*LA*_eg_ [dB(A)]	18/1 H	18/2 H	-	-	-	18/6 L	18/7 H	-	-	-	-	-	18/13 H	18/14 H
	19	*R*_w,f_ [dB(A]]	19/1 H	19/2 H	-	-	-	19/6 L	19/7 H	-	-	-	-	-	-	-
	20	*L*_w_ [dB(A]]	20/1 H	20/2 H	-	-	-	20/6 L	20/7 H	-	-	-	-	-	-	-
	21	*R*_w_ [dB(A]]	21/1 H	21/2 H	-	-	-	21/6 L	21/7 H	-	-	-	-	-	-	-
	22	*T*_rev_ [s]	22/1 VL	22/2 VL	-	-	-	22/6 VL	22/7 VL	-	-	-	-	-	-	-
Ergonomics and Universal Design	23	Dimension, Location, Dynamic, Static Aspects	23/1 H	23/2 H	-	-	-	23/6 VL	23/7 M	23/8 M	23/9 M	23/10 M	23/11 M	23/12 H	23/13 H	23/14 H

Note: Marked cell 3/12 presents identified interaction between operative temperature, *T*_o_ (i.e., thermal comfort parameter) and surface temperature of the heating system, *T*_surf_ (i.e., parameter related to heating system). Thus, operative temperature takes into account not only the temperature of the indoor air, but also the temperature of interior surfaces (i.e., heating/cooling device, constructional complexes). Additionally, marked cell 3/1 presents identified interaction between operative temperature and interior surface temperature of constructional complexes (i.e., walls, floor, and ceiling). Therefore, an insufficiently designed exterior wall might result in lower surface temperatures during winter and higher surface temperatures during summer, which consequently causes unfavourable operative temperatures and uncomfortable thermal conditions. The defined interactions present a tool for problem solving for defined effective measures towards attaining a healing environment. For example, the defined measures for cells 3/1 and 3/12 present thermally well-insulated building envelope with installation of large surface heating and cooling radiation systems that result in dynamically regulated operative temperature. Abbreviations: *C*, heat capacity of material; Clo, clothing insulation; CO_2_, carbon dioxide; CS, circadian stimulus; CH_2_O, formaldehyde; *DF*, daylight factor; *DGP*, daylight glare probability; *E*_h_, horizontal illumination; *E*_v_, vertical illumination; *g*, total solar energy transmittance; *LA*_eg_, equivalent continuous sound level; *L*_w_, weighted impact sound pressure level of indoor building elements; Met, metabolic rate; MS, melatonin suppression; *RH*_ai_, indoor relative humidity; *R*_w_, weighted sound reduction index of indoor elements; *R*_w,f_, weighted sound reduction index of facade; *SHGC*, solar heat gain coefficient; *T*_ai_, indoor air temperature; *T*_CP_, color temperature; *T*_medium_, temperature of medium; *T*_mr_, mean radiant temperature; *T*_o_, operative temperature; *T*_rev_, reverberation time; *T*_surf_, surface temperature; *U*_o_, uniformity ratio; *U*_tr_, thermal transmittance of transparent parts of building envelope; *U*_nt_, thermal transmittance of non-transparent parts of building envelope; *ν*, thermal damping factor; *η*, phase shift; *X*_diff_, material moisture content due to diffusivity; VOCs, volatile organic compounds; *v*_ai_, indoor air velocity; *τ*_ao_, light transmittance; VH, very high influential level; H, high influential level; M, medium influential level; L, low influential level; VL, very influential level; -, not detected influential level.

**Table 2:** Example of selection of individual characteristics and required/recommend indoor conditions with references.

Parameter	Burn Patient	Visitor, Healthcare Worker
**Individual Characteristics**		
*T_sk_*	*“…the body tries to raise the skin and core temperature by 2 °C secondary to a hypothalamic reset…”* [23] (p. 422) *“…burn patient strives for temperatures of about 38 °C…”* [23] (p. 492)	*“…after 3 h in a hot room (50 °C), skin temperature differentials amounted to only 2.5 °C (= 35 °C–37.5 °C), with an average core/surface gradient of ~1 °C. With normal clothing in a room at 15–20 °C, mean skin temperature is 32–5 °C…”* [36]
*T_cr_*	*“…in patients their core body temperature declines below 35.5 °C…”* [37] *“…in the general surgical population, approximately one half of patients in routine peri-operative thermal care develop a core body temperature of less than 36 °C during the peri-operative period, and a further one-third exhibit core temperatures of less than 35 °C…”* [38] *“…burn patients are by far the most susceptible to intra– and post–operative hypothermia, since the damaged skin is no longer able to prevent the loss of body heat…”* [38] *“…core temperature is generally expected to be 0.5 °C higher than body surface temperatures…”* [23] (p. 530) *“…in normal individuals the threshold range is generally near 36.5 °C–37.5 °C.”* *“In patient the threshold set point is higher and the increase is proportional to the size of the burn, 0.003 °C/% total body size area regarding the size of the burns…”* [23] (p. 205) *“… hypothermia of less than 35 °C occurs in 89% of the total operations performed in extensively burned patients…”* [39] *“…hypothermia is a particular hazard in children, with their relatively larger surface area, and in all patients with extensive burns* [23] (p. 94)	*“… the normal range for body temperature is 36.1–37.8 °C …”* [40,41]
Metabolic Rate	*“…numerous recent reports using indirect calorimetry document metabolic rates, which are 120–150% of normal* [42] (p. 399) *“…increased metabolic rate takes place after thermal injury. Within the range of 70–80% of TBSA burn injury the hypermetabolism tends to be proportional to the size of burn wound…”* [23] (p. 205) *“…using indirect calorimetry in acute patient with major burn injuries that are treated according to current standards, resting energy expenditures 110–150% above predicted values are frequently measured…”* [23] (p. 205) *“REE in adults might be 200–300% greater than predicted basal values”* [42]	*“standing relaxed 1 met, standing under stress 2 met”* [43,44,45,46,47]
Metabolism	*“Metabolic rate is increased after burn injury up to about 150% of normal levels when burn size is greater than 20–30% TBSA”* [42] *“The increase in metabolic rate approaches twice the normal”* [48] *“…metabolic rate was increased by a factor of 1.5 times basal metabolic rate…”* [49] *“…measured energy expenditure reached 2.7 ± 0.9 times the basal energy expenditure in extensively burned patients with hypothermia of less than 35 °C…”* [39]	
Effective Clothing Insulation	*“…naked 0 clo…”* [43] *“…artificial skin on very large burns covered over 80% of TBSA…”* [23] (p. 6)	*“… the insulation of different sets varies within the range of 0.54 ± 0.01 clo to 0.95 ± 0.01 clo…”* [44,45,46,47], [50] (pp. 40–46)
**Required/Recommended Indoor Environmental Conditions**		
*T*_ai_ *RH*_ai_	*“…ambient temperature and humidity should be maintained at 30–33 °C and 80%, respectively, in order to decrease energy demands and evaporative heat losses…”* [23] (p. 492) *“…the hypermetabolic response may be reduced by warming the ambient temperature to thermal neutrality (33 °C), at which point the heat for evaporation is derived from the environment, taking the burden away from the patient…”* [23] (p. 425) *“… patients need a hot environment and high relative humidity. A ward for severe burn victims should have temperature controls that permit adjusting the room temperature up to 32 °C db and relative humidity up to 95%…”* [9] *“Patients can be treated at ambient temperatures of 32–35 °C in the intensive care room with a specially designed airflow system…”* [51]	*ANSI/ASHRAE Standard 55* [34] *recommends that the relative humidity in occupied spaces is controlled in the ranges from 30% up to 60% and at air temperatures between 20 °C and 25 °C*.

Abbreviations: db, dry-bulb; REE, resting energy expenditure; *RH*_ai_, relative humidity of indoor air; *T*_ai_, room air temperature; TBSA, percentage of the total body surface area that is affected by a burn; *T*_cr_, body core temperature; *T*_sk_, skin temperature.

**Table 3:** Exergy balances for burn patient exposed to conditions created with the conventional system and UCCPS.

*T*_set up_	Input Exergy Rates [W/m^2^]				Stored [W/m^2^]	Output Exergy Rates [W/m^2^]			HbExC Rate [W/m^2^]
	Met th	C/W Rad	C/W Conv	Breath Air		C/W Rad	C/W Conv	Exhal Sweat	
**Conventional System**									
*T*_ai_ = 32 °C *T*_mr_ = 32 °C	2.29	C = 0 W = 0	0	0	0.006	C = 0 W = 0.12	C = 0 W = 0.32	0.05	1.79
**UCCPS**									
*T*_ai_ = 35 °C *T*_mr_ = 31 °C	1.30	C = 0.07 W = 0	0	0	0.001	C = 0 W = 0.01	C = 0 W = 0.03	0.02	1.36

Abbreviations: breath air, sum of exergies contained by the inhaled humid air; C/W conv in/out, warm/cool convective exergy absorbed by/discharged from the whole skin and clothing surfaces into the surrounding air; C/W rad in/out, warm/cool radiant exergy absorbed by/discharged from the whole skin and clothing surfaces; exhal sweat, exhalation and evaporation of sweat; hbExC, human body exergy consumption valid for thermoregulation; met th, metabolic thermal exergy, stored, stored exergy in the core and in the shell; *T*_set up_, set up temperature; *T*_ai_, room air temperature; *T*_mr_, mean radiant temperature; C/W, cool/warm exergy; UCCPS, user-centred cyber-physical system.

**Table 4:** Exergy balances for burn patient exposed to conditions created with the UCCPS.

*T*_set up_	Input Exergy Rates [W/m^2^]				Stored [W/m^2^]	Output Exergy Rates [W/m^2^]			HbExC Rate [W/m^2^]
	Met th	C/W Rad	C/W Conv	Breath Air		C/W Rad	C/W Conv	Exhal Sweat	
**UCCPS**									
*T*_ai_ = 35 °C *T*_mr_ = 31 °C	1.30	C = 0.07 W = 0	0	0	0.001	C = 0 W = 0.01	C = 0 W = 0.03	0.02	1.36
*T*_ai_ = 31 °C *T*_mr_ = 33 °C	2.66	C = 0 W = 0.03	0	0	0.007	C = 0 W = 0.19	C = 0 W = 0.50	0.06	1.93
*T*_ai_ = 26 °C *T*_mr_ = 35 °C	4.36	C = 0 W = 0.64	0	0	0.006	C = 0 W = 0.67	C = 0 W = 1.80	0.16	2.37

Abbreviations: breath air, sum of exergies contained by the inhaled humid air; C/W conv in/out, warm/cool convective exergy absorbed by/discharged from the whole skin and clothing surfaces into the surrounding air; C/W rad in/out, warm/cool radiant exergy absorbed by/discharged from the whole skin and clothing surfaces; exhal sweat, exhalation and evaporation of sweat; hbExC, human body exergy consumption valid for thermoregulation; met th, metabolic thermal exergy, stored, stored exergy in the core and in the shell; *T*_set up_, set temperature; *T*_ai_, room air temperature; *T*_mr_, mean radiant temperature; C/W-cool/warm exergy; UCCPS, user-centred cyber-physical system.

## Abbreviations

-	Not detected influential level
AC	Air-conditioning
breath air	Sum of exergies contained by the inhaled humid air
BRI	Building-related illness
C	Heat capacity of material
C/W conv in/out	Warm/cool convective exergy absorbed by/discharged from the whole skin and clothing surfaces into the surrounding air
C/W rad in/out	Warm/cool radiant exergy absorbed by/discharged from the whole skin and clothing surfaces; exhal sweat, exhalation and evaporation of sweat
C/W	Cool/warm exergy
CH_2_O	Formaldehyde
Clo	Clothing insulation
CO_2_	Carbon dioxide
*C*_${\mathrm{CO}}_{2}$_	Concentration of CO_2_
*C*_heat_	Energy use for heating
*C*_cool_	Energy use for cooling
CS	Circadian stimulus
db	Dry bulb
DF	Daylight factor
*DGP*	Daylight glare probability
*E*_h_	Horizontal illumination
*E*_v_	Vertical illumination
*g*	Total solar energy transmittance
H	High influential level
hbExC	Human body exergy consumption valid for thermoregulation
HVAC	Heating, ventilation and air-conditioning systems
IEQ	Indoor environmental quality
*Il*_in1_	Internal work plane illumination 1
*Il*_in2_	Internal work plane illumination 2
*Il*_out_	External illumination
*I*_rgo_	Direct solar radiation
*I*_rdo_	Reflected solar radiation
L	Low influential level
LowEx	Low exergy
$\dot{L}$	Rate of thermal load on the body surface area
*LA*_eg_	Equivalent continuous sound level
*L*_w_	Weighted impact sound pressure level of indoor building elements
M	Medium influential level
met th	Metabolic thermal exergy, stored, stored exergy in the core and in the shell
Met	Metabolic rate
MS	Melatonin suppression
*P*_e_	Precipitation detection
REE	Resting energy expenditure
*RH*_ai_	Relative humidity of indoor air
*RH*_ao_	Relative humidity of outdoor air
*R*_w_	Weighted sound reduction index of indoor elements
*R*_w,f_	Weighted sound reduction index of facade
SHGC	Solar heat gain coefficient
*T*_ai_	Temperature of indoor air
*T*_ao_	Temperature of outdoor air
TBSA	Percentage of the total body surface area that is affected by a burn
*T*_CP_	Colour temperature
*T*_cl_	Clothing temperature
*T*_cr_	Body core temperature
*T*_medium_	Temperature of medium
*T*_mr_	Mean radiant temperature
*T*_o_	Operative temperature
*T*_rev_	Reverberation time
*T*_set up_	Set temperature
*T*_sk_	Skin temperature
*T*_surf_	Surface temperature
SBS	Sick building syndrome
UCCPS	User-centred cyber-physical system
*U*_nt_	Thermal transmittance of non-transparent parts of building envelope
*U*_o_	Uniformity ratio
*U*_tr_	Thermal transmittance of transparent parts of building envelope
*v*_ai_	Indoor air velocity
VH	Very high influential level
VL	Very influential level
VOCs	Volatile organic compounds
*W*_p_	Wind speed
*W*_d_	Wind direction
*X*_diff_	Material moisture content due to diffusivity
*η*	Phase shift
*ν*	Thermal damping factor
*τ*_ao_	Light transmittance
